# Supplementary material for: A chiral molecular propeller designed for unidirectional rotations on a surface
Source: Nat Commun. 2019 Aug 20;10:3742. doi: 10.1038/s41467-019-11737-1 (PMC6702202; doi:10.1038/s41467-019-11737-1)
Supplement: Supplementary file 1 — Supplementary Information [file 41467_2019_11737_MOESM1_ESM.pdf]

Supplementary Information

A Chiral Molecular Propeller Designed for Unidirectional Rotations on a Surface

Yuan Zhang[1], Jan Patrick Calupitan[2,3,4], Tomas Rojas[5,6], Ryan Tumbleson[6], Guillaume Erbland[4], Claire Kammerer[4], Tolulope Michael Ajayi[6], Shaoze Wang[6], Larry A. Curtiss[5], Anh T. Ngo[5], Sergio E. Ulloa[6], Gwénaël Rapenne[*2,3,4], and Saw Wai Hla[*1,6]

[1]*Center for Nanoscale Materials, Argonne National Laboratory, Lemont, IL 60439, USA*

[2]*Division of Materials Science, Nara Institute of Science and Technology, NAIST, 8916-5 Takayama-cho, Ikoma, Nara 630-0192, Japan*

[3]*International Collaborative Laboratory for Supraphotoactive Systems, Naist-CEMES, CNRS UPR 8011, 29 rue J. Marvig, F-31055 Toulouse, France*

[4]*CEMES, Université de Toulouse, CNRS, Toulouse, France*

[5]*Materials Science Division, Argonne National Laboratory, Lemont, IL 60439, USA*

[6]*Nanoscale and Quantum Phenomena Institute and the Department of Physics and Astronomy, Ohio University, Athens, OH 45701, USA*

**Corresponding authors: gwenael-rapenne@ms.naist.jp, hla@ohio.edu*

Table of Contents

- **Supplementary Figure 1.** Phenyl rings tilt angles in the molecular gear
- **Supplementary Figure 2.** Phenyl rings tilt angles in the stator only structure
- **Supplementary Figure 3.** Different propeller conformations
- **Supplementary Method 1.** Electric field energy stored in the molecular propeller
- **Supplementary Table 1.** Threshold electric field and electrical energy for rotation
- **Supplementary Figure 4.** LUMO orbital and IET threshold bias
- **Supplementary Discussion 1.** Distinguishing different STM manipulation modes
- **Supplementary Figure 5.** Forward and reverse mechanical rotations
- **Supplementary Figure 6.** Rotation with load
- **Supplementary References**

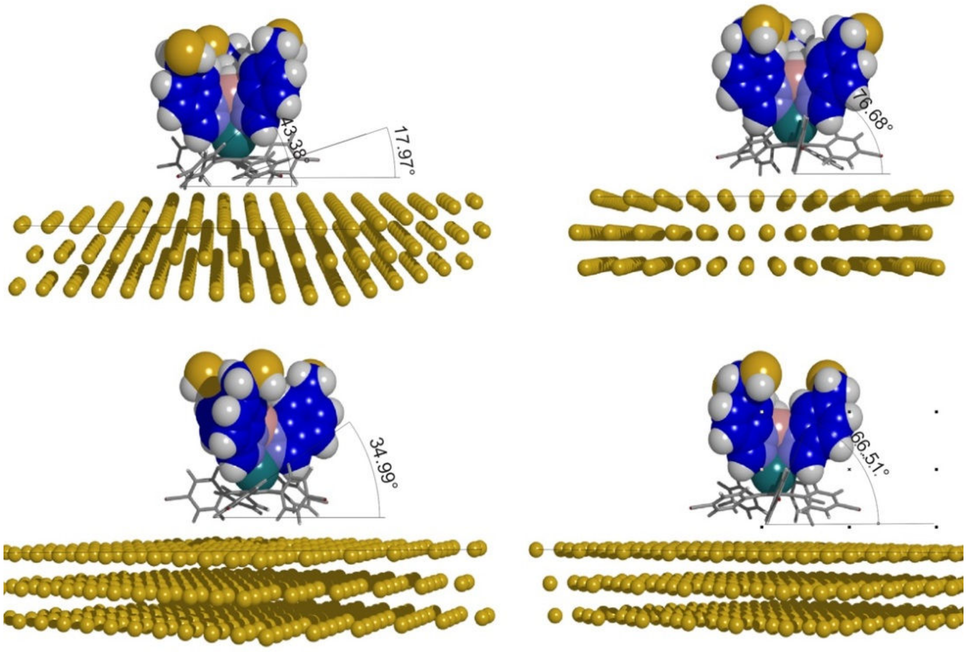

Supplementary Figure 1. The tilt angles of the phenyl rings from the stator are measured as 17.97°, 43.88°, 76.68°, 34.99°, and 66.51° from the geometrically relaxed DFT calculations for the molecular propeller adsorbed on a three-layer Au(111) slab.

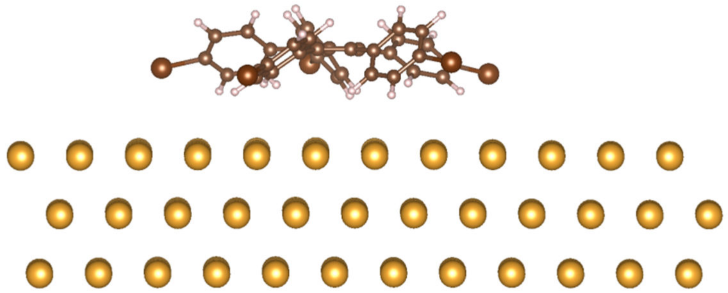

Supplementary Figure 2. The DFT calculated structure of the stator only adsorption on Au(111), which gives the phenyl ring tilt angle ~39°.

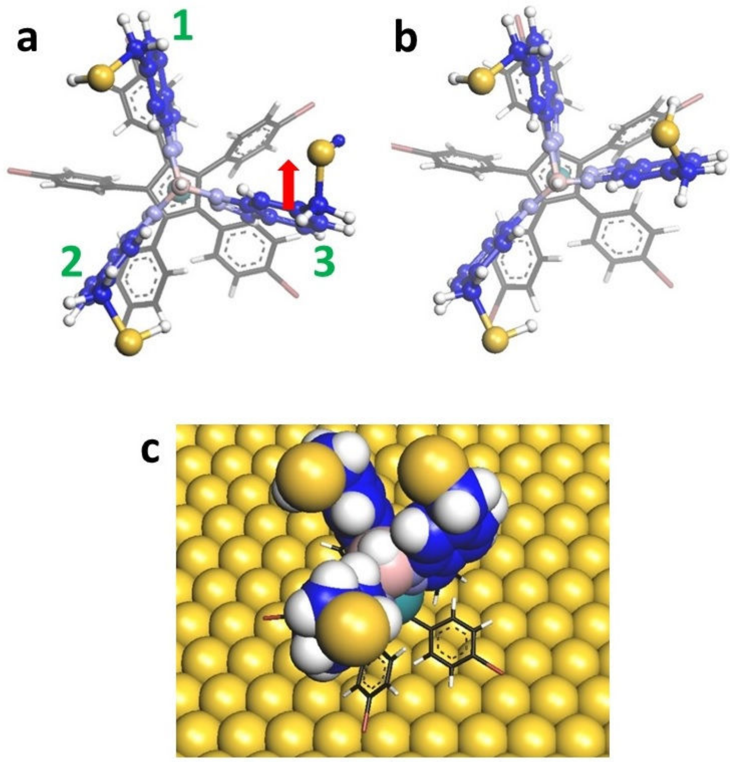

Supplementary Figure 3. (**a**) The energy minimum configuration of the molecular propeller resulted from the geometrically relaxed DFT calculations. Here, the blade '3' is located between two phenyl rings of the stator, and it has a free space to move forward as shown with the red arrow. By keeping the blade '1' and '2' in their positions while moving the blade 3 forward along the red arrow in (**a**), a new conformation with asymmetric propeller angles is formed (**b**). This new configuration has only slightly higher energy (45 meV) over the energetic minimum position shown in (**a**). Surface atoms are omitted in the plots (a) and (b) for the clarity. (**c**) The same configuration shown in (**b**) with the surface atoms included.

Supplementary Method 1. Electric field energy stored in the molecular propeller

The electric field energy stored in the molecular propeller is calculated for three threshold electric field values and summarized in the Supplementary Table 1. The calculations are performed for the molecule under an external electric field for a field range of 0.2 V/Å to 0.3 V/Å in the direction perpendicular to the plane of the stator (the z-axis). The structure optimized at the zero-field is used to investigate the energetic changes under the applied perpendicular electric field. The approach developed by Neugebauer and Scheffler, as implemented in VASP [1], is used to introduce the external field in the unit cell. In this approach, the electric field is introduced by an artificial dipole sheet in the vacuum region of the cell. To avoid interactions between periodic images, a dipole potential correction is included [2].

Supplementary Table 1. Threshold electric field and electrical energy for rotation

Threshold Electric Field (V/ Å)	Energy (eV)
0.30	-0.89
0.25	-0.66
0.20	-0.51

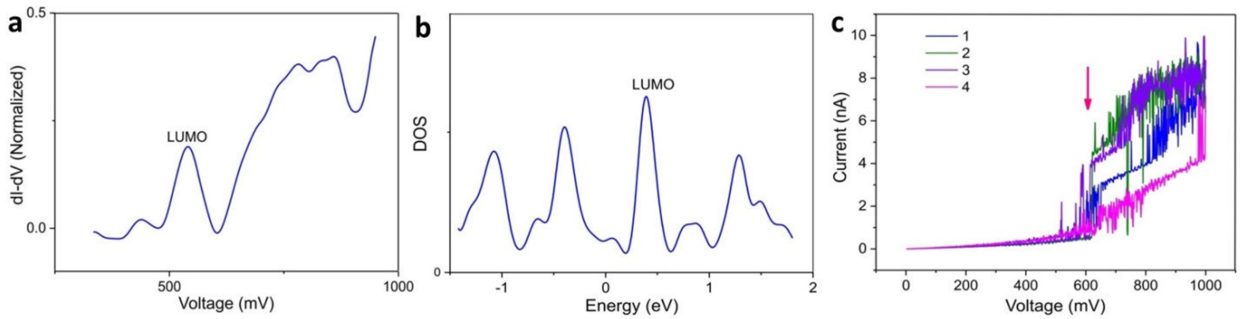

Supplementary Figure 4. (**a**), Normalized dI/dV [(dI/dV)/(I/V)] as a function of tunneling bias reveals the LUMO at ~0.55 V. (**b**) Projected DOS of the molecular propeller on Au(111) as a function of energy calculated by DFT. The LUMO orbital is indicated. (**c**) Four consecutive I-V measurements shows abrupt changes in current ~0.6 V (indicated with an arrow) due to IET induced rotations of the propeller. From such I-V measurements, the threshold bias for the IET induced rotations is determined as ~0.6 V.

Supplementary Discussion 1. Distinguishing different STM manipulation modes

In an STM application, tunneling current, electric field and tip-sample interactions simultaneously exist at the tip-sample junction. However, STM manipulation modes can be selectively turned on by controlling specific tunneling parameters. For an inelastic electron tunneling (IET) induced manipulation, two parameters are the key: 1). The probability of tunneling electron energy transfer, and 2). the energy of tunneling electrons. The probability of tunneling electron energy transfer depends on the current. The quantum yield [3] for a similar molecule has been determined [4] as ~2.2×10^{-10}. Here, the quantum yield Y = Re/I, where R is the rate of energy transfer, 'e' is the electronic charge, and 'I' is the tunneling current. For an estimated rate of about 1/s, the minimum current of ~1 nA is required to trigger the IET process to rotate the molecular propeller. For the second requirement, the energy of the tunneling electron needs to exceed the energetic location of unoccupied molecular orbitals. From the dI/dV curve, the LUMO energy is determined as ~0.55 eV (Supplementary Fig. 4a). This energy value is in relative agreement with the DFT result (Supplementary Fig. 4b).

Supplementary Fig. 4c shows the threshold energy measurement where the I-V curves exhibit abrupt changes in the currents at ~0.6 V caused by the rotation of the propeller blades. This energy is within the range of LUMO energy and therefore it is attributed as caused by the IET induced rotation via a temporary electron attachment to the LUMO orbital. Moreover, from the tunneling resistance of 0.3 GΩ (derived from the V_t = 0.6 V, and I_t = 2nA) generally used for IET rotations here, a relative tip-sample distance of ~6Å is determined, and an electric field of ~0.1 V/Å is estimated. This value is much lower than the critical electric field of 0.25 V/Å required for the electric field induced rotation of the molecular propeller. Thus, IET process is the key for the observed rotations.

For the force induced rotations of the propellers, a tunneling bias range of 0.01 V to 0.1 V is used. The estimated electric field strength here is from 0.004 V/Å to 0.02 V/Å, and therefore the contribution of the electric field effect can be ruled out. This is further supported by the fact that the manipulation signals mostly exhibit pushing curves indicating that the tip-sample mechanical contact is the key for the force induced manipulations.

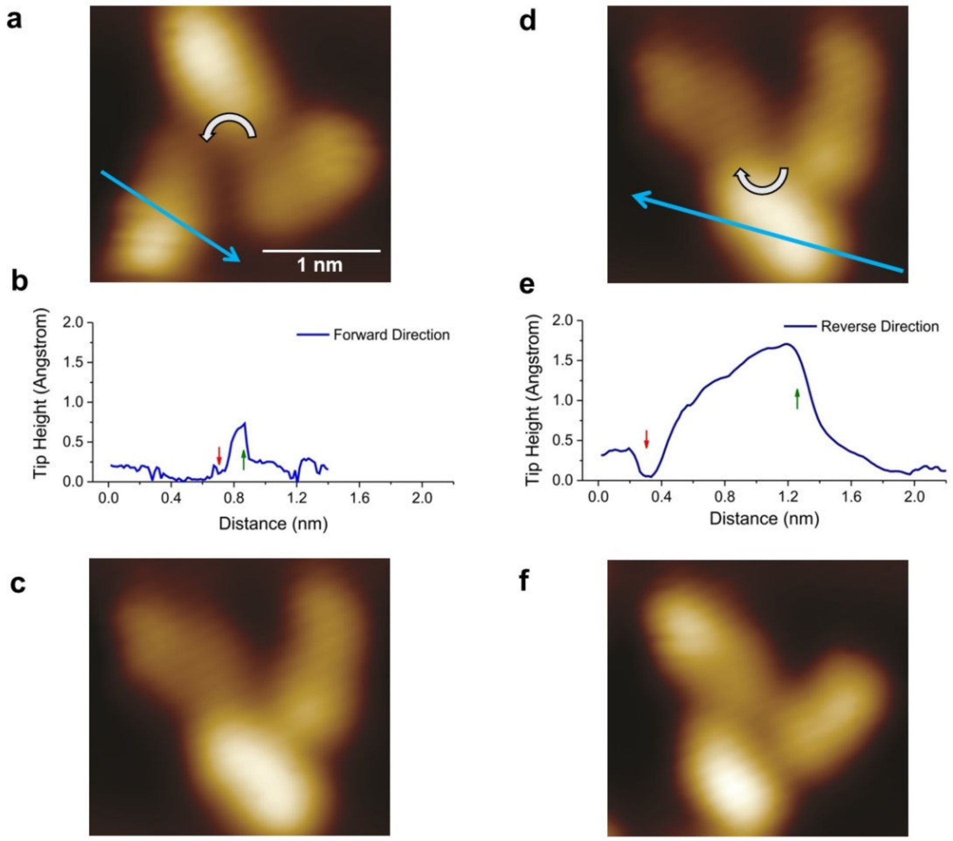

Supplementary Figure 5. (**a**) STM image of a 'P' type molecular propeller is rotated to anticlockwise direction by pushing a lower blade with the STM tip along the blue arrow. (**b**) The corresponding manipulation signal shows a dip (indicated with a red arrow) following a pushing like signal (green arrow). (**c**) The STM image acquired after the manipulation shows the molecule is rotated anticlockwise direction for 41°. (**d**) For the reverse rotation process, the STM tip is lowered for 4 Å from its initial tip-height as before, and then move along the blue arrow direction. (**e**) The corresponding manipulation signal again reveals a dip (red arrow) followed by a pushing like signal (green arrow). Notice that the upslope in this manipulative curve is much larger than previous rotation event. The higher the upslope curve, the larger the contact area between the tip and the propeller and accordingly, the pushing force becomes larger. (**f**) The STM image acquired after this manipulation shows that the propeller is rotated 21° clockwise. Although a much larger pushing force is required for the reverse rotation, the propeller is rotated to a smaller angle as compared to the forward rotation. [STM imaging parameters for (**a**), (**c**), (**d**), and (**f**): V_t = 1 V, I_t = 50 pA, manipulation parameters for (**b**), and (**e**): V_t = 0.1 V, R_t = 1.3 MΩ].

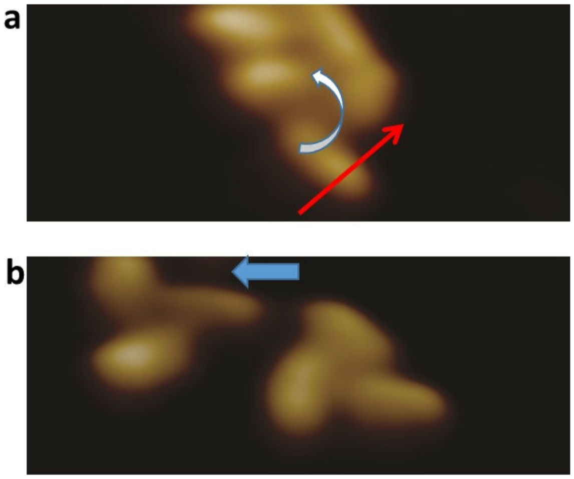

Supplementary Figure 6. (**a**) STM image showing two coupled molecular propeller. The lower propeller is rotated anti-clockwise direction by the tip moving a straight line along the red arrow direction. (**b**) STM image after rotation of the lower propeller shows that the upper propeller is thrown out towards the direction indicated with an arrow. Moving the upper propeller here is due to the steric repulsion between the propeller blades of the two molecules, and this demonstrates that the propeller can be used to move molecular load [R_t =1 MΩ, Image parameters: 7.5 x 3 nm^2, V_t = -2.0 V, I_t = 33 pA].

References

[1] Neugebauer, J., & Scheffler, M. Adsorbate-substrate and adsorbate-adsorbate interactions of Na and K adlayers on Al(111). *Phys. Rev. B* **46**, 16067-16080 (1992).

[2] Bengtsson, L. Dipole correction for surface supercell calculations. *Phys. Rev. B* **59**, 12301-12304 (1999).

[3] Iancu, V., & Hla, S.-W. Realizing of a four-step molecular switch in scanning tunneling microscope manipulation of single chlorophyll-a molecules. *Proc. Nat. Acad. Sci.* **103**, 13718-13721 (2006).

[4] Perera, U. G. E., Ample, F., Kersell, H., Zhang, Y., Vives, G., Echeverria, J., Grisolia, M., Rapenne, G., Joachim, C., & Hla, S.-W. Controlled clockwise and anticlockwise rotational switching of a molecular motor. *Nat. Nanotechnol.* **8**, 46-51 (2013).
