# Supplementary material for: A chiral molecular propeller designed for unidirectional rotations on a surface
Source: Nat Commun. 2019 Aug 20;10:3742. doi: 10.1038/s41467-019-11737-1 (PMC6702202; doi:10.1038/s41467-019-11737-1)
Supplement: Supplementary file 3 — Description of Additional Supplementary Files [file 41467_2019_11737_MOESM3_ESM.pdf]

**Description of Additional Supplementary Files**

File Name: Supplementary Movie 1
Description: Pi-ring tilt angle during rotation

File Name: Supplementary Movie 2
Description: Propeller rotation movie
