# Supplementary figures and images for: A chiral molecular propeller designed for unidirectional rotations on a surface
Source: Nat Commun. 2019 Aug 20;10:3742. doi: 10.1038/s41467-019-11737-1 (PMC6702202; doi:10.1038/s41467-019-11737-1)

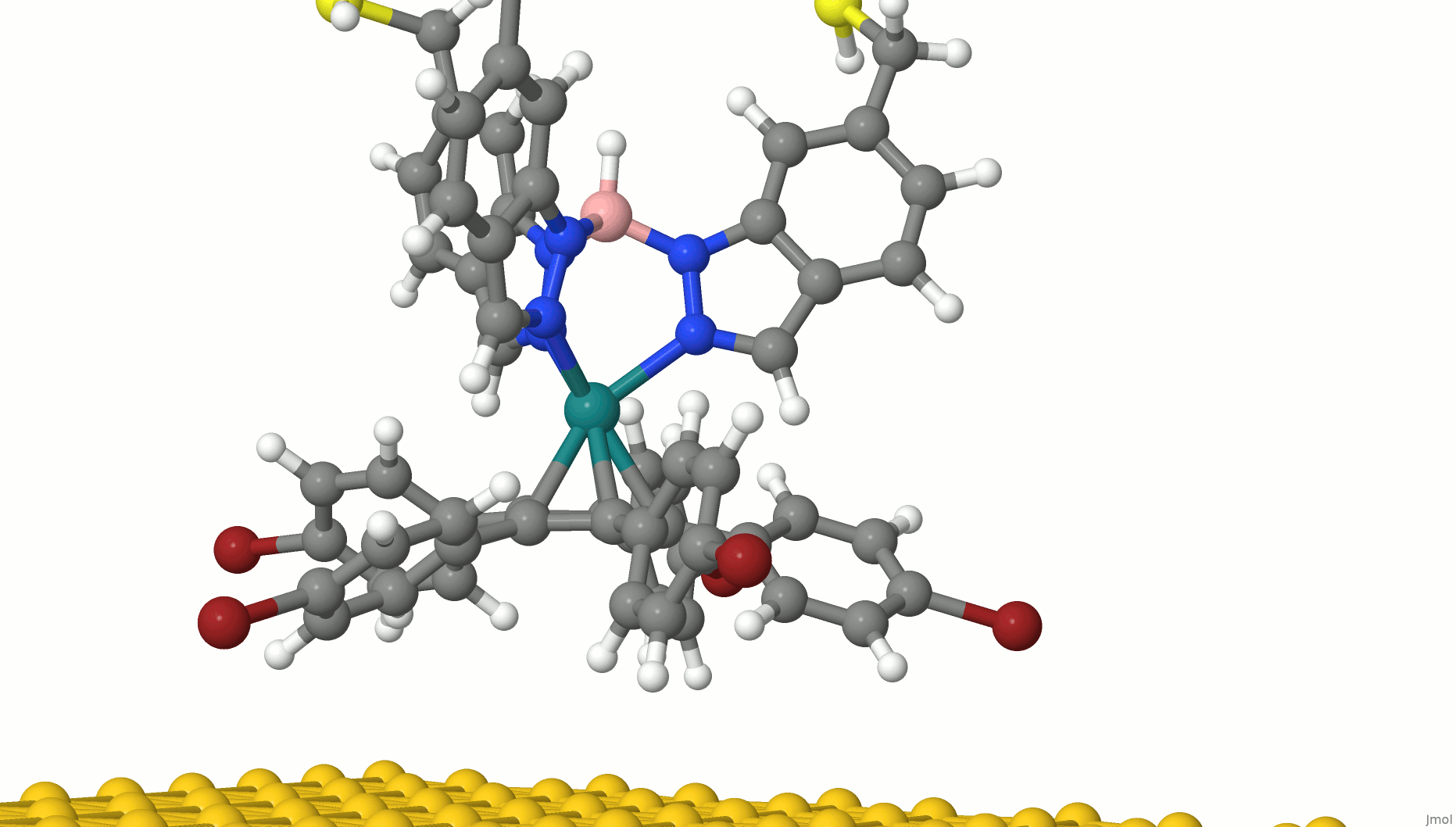

Supplement: Supplementary file 4 — Supplementary Movie 1 [file 41467_2019_11737_MOESM4_ESM.gif]
